# Validation of the Japanese version of the Central Sensitization Inventory in patients with musculoskeletal disorders

**DOI:** 10.1371/journal.pone.0188719

**Published:** 2017-12-07

**Authors:** Katsuyoshi Tanaka, Tomohiko Nishigami, Akira Mibu, Masahiro Manfuku, Satoko Yono, Yoshikazu Shinohara, Akihito Tanabe, Rei Ono

**Affiliations:** 1 Department of Rehabilitation, Tanabe Orthopaedics, Osaka, Osaka, Japan; 2 Department of Community Health Sciences, Kobe University Graduate School of Health Sciences, Kobe, Hyogo, Japan; 3 Department of Nursing and Physical Therapy, Konan Women’s University, Kobe, Hyogo, Japan; Taipei Veterans General Hospital, TAIWAN

## Abstract

**Background:**

Many musculoskeletal pain conditions are characterized by hypersensitivity, which is induced by central sensitization (CS). A questionnaire, the Central Sensitization Inventory (CSI), was recently developed to help clinicians identify patients whose presenting symptoms may be related to central sensitivity syndrome (CSS). The aims of the present study were to examine criterion validity and construct validity of the Japanese version of the CSI (CSI-J), and to investigate prevalence rates of CS severity levels in patients with musculoskeletal disorders.

**Methods:**

Translation of the CSI into Japanese was conducted using a forward-backward method. Two hundred and ninety patients with musculoskeletal pain disorders completed the resultant CSI-J. A subset of the patients (n = 158) completed the CSI-J again one week later. The relationships between CSI and clinical symptoms, EuroQol 5-dimension (EQ-5D) and Brief Pain Inventory (BPI), were examined for criterion validity. EQ-5D assesses Health-related QOL and BPI measures pain intensity and pain interference. The psychometric properties were evaluated with analyses of construct validity, factor structure and internal consistency, and subsequently investigate the prevalence rates of CS severity levels.

**Results:**

The CSI-J demonstrated high internal consistency (Cronbach’s α = 0.89) and test-retest reliability was excellent value (ICC = 0.85). The CSI-J was significantly correlated with EQ-5D (r = −0.44), pain intensity (r = 0.42), and pain interference (r = 0.48) (p < 0.01 for all). Ten percent of the participants were above the cutoff “40”. The exploratory factor analysis resulted in 5-factor model.

**Conclusions:**

This study reported that the CSI-J was a useful and psychometrically sound tool to assess CSS in Japanese patients with musculoskeletal disorders. The finding of the prevalence rates of CS severity levels in patients with musculoskeletal disorders may help clinicians to decide strategy of treatment.

## Introduction

Central sensitization (CS) is defined as increased responsiveness of nociceptive neurons in the central nervous system to normal or subthreshold afferent input by the International Association for the Study of Pain [1]; it is operationally defined as an amplification of neural signaling within the central nervous system that elicits pain hypersensitivity [2]. CS may be responsible for mechanical hyperalgesia, allodynia, and/or referred pain, which are often present in chronic pain syndromes. Musculoskeletal disorders describe a wide range of inflammatory and degenerative conditions affecting muscles, tendons, joints, and the associated areas, which form a major and increasing cause of disability [3,4]. Many chronic musculoskeletal pain conditions, such as osteoarthritis [5–7], rheumatoid arthritis [8], low back pain [6,9,10], persistent neck pain [11–14], fibromyalgia [6,15,16], and tennis elbow [17], are characterized by hypersensitivity, which is induced by CS. The nociceptive trigger is targeted for treatment in many cases of acute musculoskeletal pain [18,19], and some patients with musculoskeletal disorders complain of persistent pain despite treatment. It is estimated that 10% of the reported and persistent physical symptoms cannot be explained by organic factors in the general population [20]. Screening for the occurrence of these generalized hypersensitivities, captured as CS, is beneficial to clinicians, that is distinguishing through the Central Sensitization Inventory (CSI) enables clinicians to provide more specific treatments. Even if the duration of pain is shorter than defined as chronic pain, the screening is beneficial.

Central sensitivity syndromes (CSS) are an overlapping and similar group of syndromes that are bound by the common mechanisms of CS, which lead to hypersensitivity including hyperalgesia and allodynia. CSS conditions are not confined to one specific region of the body. This is the reason why CSS conditions are disorders of pain processing in the central nervous system. CS is likely to play a causative role in CSS, probably with other risk factors [21]. The presence of multiple CSS disorders and/or related medical conditions in the same patient was found to be associated with more limited functionality and greater disability [22]. Besides pain, many clinical symptoms, including fatigue, concentration difficulties, sleep disturbances, and nonrefreshing sleep, have been described in CSS patients [21,23,24]. The occurrence of multiple somatic symptoms is associated with poorer treatment outcomes and higher healthcare utilization [25–27].

Direct measures of CS are often conducted by Quantitative Sensory Testing (QST), which consists of static and dynamic psychophysical tests to quantify somatosensory function in response to the controlled stimuli [28]. Although many previous studies have shown a relationship between clinical symptoms and CS measured with QST [29–31], a disadvantage is the high cost of the corresponding system and therefore the reduced applicability in clinical practice. The CSI was recently developed as a comprehensive screening instrument for CS [32]. This questionnaire is designed to help clinicians identify patients whose presenting symptoms may be related to CSS. Part A of the CSI assesses 25 health-related symptoms that are common to CSSs, with total scores ranging from 0 to 100. Part B (which is not scored) asks whether one or more specific disorders, including seven separate CSSs (fibromyalgia, chronic fatigue syndrome, temporomandibular joint disorder, irritable bowel syndrome, migraine or tension headaches, multiple chemical sensitivities, and restless leg syndrome), have been diagnosed previously. The CSI demonstrates good psychometric properties, clinical utility, and initial construct validity [32]. In addition, the CSI severity level were associated with patient-reported depressive symptoms, perceived disability, sleep disturbance, and pain intensity [33]. Furthermore, CSI scores were also positively correlated with the number of diagnosed CSSs [34,35].

Translation and validation studies of the CSI have been completed in several different languages, including Dutch [36], French [37], and Spanish [38]. Tanaka et al. translated the original English version into Japanese and linguistically validated it, with the aim of introducing the CSI in Japan [39]; however, psychometric properties of the Japanese version of the CSI remain uninvestigated. In addition, while previous studies have targeted chronic pain patients, no study has addressed the cluster of musculoskeletal disorders. Therefore, the aims of the present study were to examine criterion validity and construct validity of the Japanese version of the CSI (CSI-J), and to investigate prevalence rates of CS severity levels in patients with musculoskeletal disorders.

## Methods

### Translation of the questionnaire

The Japanese version was linguistically validated through the general cross-cultural adaptation process: forward-translation, back-translation, and cognitive debriefing. First, the Japanese speakers (KT, TN, and AM) translated the original CSI items from English to Japanese. Second, the revised Japanese version was back-translated from Japanese to English by a native English speaker. Third, the back-translation was checked and approved by the developer of the original CSI, and a provisional version of the CSI-J was created. Finally, the provisional CSI-J was administered to six native Japanese patients with musculoskeletal disorders, who provided feedback on comprehensibility and completeness of the content and time exposure. This final pre-testing revealed ambiguity about the answer choices. Therefore, we altered the expression of them, and we developed a final version of the CSI-J (S1 Table) [39].

### Participants

A total of 290 patients were recruited from an orthopedic clinic, of which those who were aged between 20 and 80 years and suffered from musculoskeletal pain, such as neck, shoulder, low back, hip, knee, or ankle, were included. Exclusion criteria included patients diagnosed with cancer, brain or spinal cord injury, neurological disease, dementia, and poor Japanese language comprehension. Ethical approval was obtained from the Institutional Ethics Committee of Konan Women’s University. Informed consent was obtained from all subjects prior to the study. The study was conducted in accordance with the Declaration of Helsinki.

### Procedure

Demographic (age, gender, height, weight), CSI-J, and four pain-related outcomes [pain duration, health-related quality of life (QOL), pain intensity, and pain interference] were assessed in all participants. A test-retest reliability of the CSI-J was determined with a time interval of 1 week. These domains were selected because patients whose presenting symptoms may be related to a CSS (e.g. chronic whiplash-associated disorders, fibromyalgia, and PTSD) showed significant relationships between CS outcome and QOL, pain intensity, and disability [33, 40–42].

The CSI-J consists of two parts: A and B. Part A is a 25-item self-report questionnaire designed to assess health-related symptoms that are common to CSSs. Each item is rated on a 5-point Likert-type scale (0 = never and 4 = always), with total scores of 0–100. Part B (which is not scored) is designed to determine whether one or more specific disorders, including seven separate CSSs, have been previously diagnosed [restless leg syndrome, chronic fatigue syndrome, fibromyalgia, temporomandibular joint disorder, migraine or tension headaches, irritable bowel syndrome, multiple chemical sensitivities, neck injuries (including whiplash), anxiety or panic attacks, and depression].

Health-related QOL was measured using EuroQol 5-dimension (EQ-5D) [43]. EQ-5D was developed as an instrument that is not specific to disease, but standardized, and can be used as a complement to existing health-related QOL measures [44]. It comprises the following five dimensions: mobility, self-care, usual activities, pain/discomfort, and anxiety/depression. Each dimension has three grades (no problems, some problems, and extreme problems), which can generate a single index value for each health state. These values are numbers on a scale with 1 for full health and 0 for being dead. Tsuchiya et al. showed the Japanese value set [45].

Pain intensity and pain interference were measured using the Brief Pain Inventory (BPI) [46,47]. It consists of four pain intensity and seven pain interference items. These items were presented with 0–10 scales, with 0 = no and 10 = worst (completely). From these, individual pain intensity and pain interference scores are calculated by averaging. The validation and clinical utility of BPI has been evaluated for several disorders [48–50]. To investigate the prevalence rates of CS severity levels, we referred to the five categories with increasing severity [33]. The authors reviewed the score distributions of previously published CSI study samples, including those with no CSS diagnosis, those with a single CSS diagnosis, those with multiple CSS diagnoses, and a group of nonpatient comparison subjects. Through empirical reasoning and deduction, using these score distributions as a guide, the CSI was divided into five categories with increasing severity: subclinical (0–29), mild (30–39), moderate (40–49), severe (50–59), and extreme (60–100).

### Statistical analyses

All statistical analysis was performed using the SPSS version 22 (IBM SPSS Statistics for Windows, Version 22.0. Armonk, NY: IBM Corp.). The internal consistency of the CSI-J was assessed using Cronbach’s α. An α value between 0.70 and 0.90 was considered as good, and higher than 0.90 was considered as excellent. In addition, CSI-J reliability was assessed using scores obtained from a second round of the questionnaire, answered by participants after 1 week of their first questionnaire completion. Intraclass correlation coefficients (ICC, two-way random effect model with single measures) were calculated for examining the test-retest reliability. ICC_2,1_ values ≤0.40 were considered to indicate fair reliability, 0.41–0.60 moderate reliability, 0.61–0.80 substantial reliability, and ≥0.81 almost perfect reliability [51]. The relationships between the CSI-J score and pain intensity (BPI), pain interference (BPI), and health related QOL (EQ-5D) were examined. These associations were investigated using Spearman’s correlation coefficients. One-way analyses of variance were used to compare CSI score by number of CSS diagnoses.

While validating a new questionnaire or translated version of an existing questionnaire, it is advised to first initiate a data reduction procedure by means of an exploratory factor analysis (EFA). An EFA was conducted with the maximum likelihood method using a promax rotation. Factors were considered for eigenvalues >1 [36, 38, 52–55]. The cut-off for the loadings was set at 0.40.

## Results

### Sample characteristics

Table 1 provides a summary of the demographic characteristics and clinical profile of all participants. In total, the mean score of CSI-J was 21.91 ± 13.31 (mean, SD).

**Table 1:** Characteristics of participants.

	Mean (SD) or N (%)
Demographic information	
Age (years)	51.14 (15.61)
Gender (female)	188 (64.83)
Height (cm)	162.00 (9.08)
Weight (kg)	58.90 (11.92)
Clinical status	
Duration of Pain (weeks)	21.00 (47.28)
Central Sensitization (CSI-J, 0–100)	21.91 (13.31)
Health-related QOL (EQ-5D, 0–1)	0.713 (0.124)
Pain intensity (BPI, 0–10)	2.93 (1.82)
Pain interference (BPI, 0–10)	2.53 (2.19)

CSI-J: Japanese version of the Central Sensitization Inventory; EQ-5D: EuroQol 5-dimension; BPI: Brief Pain Inventory.

### The internal consistency and test-retest reliability

The CSI-J showed a high degree of internal consistency (Cronbach’s α = 0.89) with an individual item range from 0.88 to 0.89.

Of the all participants, 158 patients answered the questionnaire twice. There was an excellent agreement between the test and retest total scores, with an ICC_2,1_ of 0.85 [95% confidence interval (CI) 0.80–0.89]. An analysis of individual item scores revealed that 21 of 25 items showed an ICC >0.60 (range 0.61–0.82). Items 2 (0.48), 3 (0.48), 11 (0.57), and 19 (0.38) showed an ICC <0.60.

### Correlation with clinical symptoms

While the CSI-J was not related with the duration of pain (r = 0.10, p = 0.11), it was significantly correlated with EQ-5D (r = −0.44), pain intensity (r = 0.42), and pain interference (r = 0.48) (p < 0.01 for all, Table 2).

**Table 2:** Results of bivariate correlations between CSI-J and clinical symptoms.

Variance	Correlation coefficient	p-value
Health-related QOL (EQ-5D)	-0.44	< 0.01
Pain intensity (BPI)	0.42	<0.01
Pain interference (BPI)	0.48	<0.01
Duration of pain	0.10	0.11

EQ-5D: EuroQol 5-dimension; BPI: Brief Pain Inventory.

### Prevalence

Of the 290 patients, 214 patients (73.79%) indicated subclinical, 44 patients (15.17%) indicated mild, and 32 patients (11.00%) indicated moderate or higher severity. No patients had been diagnosed with fibromyalgia and multiple CSs (Table 3). As shown in Table 4, of the total 290 patients, 81 (27.93%) patients were diagnosed with CSS. Patients diagnosed with only one CSS (26.44 ± 11.47; 95% CI, 23.55–29.33) or 2 or more CSSs (32.50 ± 16.46; 95% CI, 24.31–40.69) scored higher on the CSI than those with no CSS diagnosis (19.64 ± 12.81; 95% CI, 17.90–21.39; p < 0.01).

**Table 3:** Prevalence rates of CS severity levels and frequency of diagnoses.

	N (%)
CSI-J score	
Subclinical (0–29)	214 (73.79)
Mild (30–39)	44 (15.17)
Moderate (40–49)	21 (7.24)
Severe (50–59)	8 (2.76)
Extreme (> 60)	3 (1.03)
Diagnoses	
Restless leg syndrome	1 (0.34)
Chronic fatigue syndrome	1 (0.34)
Fibromyalgia	0 (0)
Temporomandibular joint disorder	21 (7.24)
Migraine or tension headaches	26 (8.97)
Irritable bowel syndrome	8 (2.76)
Multiple chemical sensitivities	0 (0)
Neck injury (including whiplash)	20 (6.90)
Anxiety or panic attacks	11 (3.79)
Depression	17 (5.86)

CSI-J: Japanese version of the Central Sensitization Inventory.

**Table 4:** Comparison among No CSS patients and CSS patients.

	Mean (SD)	95%CI
No CSS (N = 209, 72.07%)	19.64 (12.81)	17.90–21.39
1 CSS (N = 63, 21.72%)	26.44 (11.47) **	23.55–29.33
2+ CSS (N = 18, 6.21%)	32.50 (16.46) **	24.31–40.69

CSS: Central sensitivity syndrome; **: significant difference with No CSS, p < 0.01; CI: confidence interval.

### Exploratory factor analysis

The EFA produced a five-factor model, of which three factors “Emotional distress”, “Urological and general symptoms”, and “Headache/Jaw symptoms” were similar in comparison to the categorization of items in the original English version [32]. Factor 1, named “Emotional distress”, encompassed four items (items 15, 16, 17, and 24) pertaining to “Emotional distress” from the original article. Items 3, 13, and 23 did not load on this factor in the present study. Factor 2, named “Urological and general symptoms”, in the present study encompassed three items (items 11, 21, and 25) pertaining to “Urological symptoms” from the original article. Items 9, 22, and 23 loaded sufficiently high on this factor in the present study. In addition, factor 2 encompassed item 9 (“Pain all over the body”), which refers to a general problem and pertains to the “General disability and physical symptoms” category in the Dutch version [36]. As items 2 (“Muscles stiff/achy”) and 18 (“Tension neck and shoulder”) both referred to muscle problems, this factor was named “Muscle symptoms”. “Headache/Jaw symptoms” shared three items (items 4, 10, and 19) with the same factor in the original article. Although items 7 (“Sensitivity to bright lights”) and 20 (“Certain smells produce dizziness”) did not load on this factor, in contrast to the original article, naming this factor as “Headache/Jaw symptoms” seems adequate. “Sleep disturbance” is a unique factor compared with the original and the Dutch versions. This factor encompassed three items (item 1, “Unrefreshed in morning”; item 8, “Easily tired with physical activity”; and item 12, “Do not sleep well”), all of which relate to sleep problems. The factor loading of items 3, 5, 6, 7, 13, 14, and 20 was <0.40 (Table 5). All interfactor correlation coefficients after promax rotation indicated positive correlations (Table 6).

**Table 5:** Factor loadings of the exploratory factor analysis with promax rotation.

Item No.		F1	F2	F3	F4	F5	Not loading
**1**	Unrefreshed in morning	-.01	-.15	**.52**	-.03	**.54**	
**2**	Muscles stiff/achy	-.04	.13	**.75**	-.10	-.06	
**3**	Anxiety attacks	.30	.32	.17	-.01	-.08	X
**4**	Grind/clench teeth	.14	-.08	-.02	**.45**	.07	
**5**	Diarrhea/constipation	.02	.07	.04	.05	.40	X
**6**	Need help with daily activity	.14	.29	.09	-.02	.01	X
**7**	Sensitive to bright light	.20	.09	-.10	.17	.24	X
**8**	Easily tired with physical activity	.14	.24	.06	.03	**.42**	
**9**	Pain all over body	-.15	**.51**	.23	.17	.02	
**10**	Headaches	-.03	-.04	.14	**.57**	.23	
**11**	Bladder/urination pain	.01	**.50**	-.12	.14	.08	
**12**	Do not sleep well	.04	.01	.31	.02	**.45**	
**13**	Difficulty concentrating	.38	.21	-.01	-.06	.28	X
**14**	Skin problems	.05	.09	-.12	.19	.24	X
**15**	Stress makes symptoms worse	**.52**	-.09	.06	.18	.20	
**16**	Sad or depressed	**1.03**	-.05	.02	.07	-.17	
**17**	Low energy	**.81**	-.03	-.01	-.06	.14	
**18**	Tension neck and shoulder	-.05	-.03	**.49**	.25	.01	
**19**	Pain in jaw	.05	.27	.06	**.48**	-.13	
**20**	Certain smells make dizzy	.09	-.06	-.17	.29	.35	X
**21**	Urinate frequently	-.16	**.75**	-.08	.01	.09	
**22**	Restless legs	.13	**.50**	.14	.01	-.02	
**23**	Poor memory	.17	**.41**	-.21	-.17	.28	
**24**	Trauma as a child	**.40**	.03	-.14	.09	.07	
**25**	Pelvic pain	.08	**.46**	.18	-.11	-.05	

**Table 6:** Promax factor correlations of the Japanese CSI.

	Factor 1	Factor 2	Factor 3	Factor 4	Factor 5
**Factor 1**	-				
**Factor 2**	.54	-			
**Factor 3**	.39	.43	-		
**Factor 4**	.39	.36	.43	-	
**Factor 5**	.66	.50	.41	.40	-

## Discussion

The aim of this study was to validate and reveal the underlying structure of the CSI-J in a sample of Japanese patients with musculoskeletal disorders. Our results showed that the CSI-J had excellent internal consistency and test-retest reliability, as well as significant positive associations with two pain-related scales of BPI. Additionally, there was a significant negative association between the CSI-J and EQ-5D. Factor analysis revealed that the CSI-J had a 5-factor structure, which contrasts with the English [32], Dutch [36], Spanish [38].

The internal consistency of the CSI-J was 0.89, below the accepted 0.95 threshold for item redundancy [56]. It was consistent with the English (Cronbach’s α = 0.87) [32], Dutch (Cronbach’s α = 0.91) [36], and Spanish versions (Cronbach’s α = 0.87) [38], indicating that the CSI-J remains stable in different cultures.

The ICC score was 0.85, indicating that the CSI-J has excellent reliability, corroborating earlier reports on the English (0.82) [32], Dutch (0.88) [36], and French (0.91–0.94) [37] versions. Therefore, the results of the present study revealed that the CSI-J is a reliable instrument. Furthermore, we found significant positive associations between the CSI-J and the two pain scales of the BPI, pain intensity, and pain interference, and significant negative associations between CSI-J and health-related QOL. The findings support the psychometric soundness of the CSI-J.

The EFA yielded a 5-factor model, which contrasts with the English [32], Dutch [36], and Spanish versions [38], but the French version [37] is consistent with the present study. The English and Dutch version revealed a 4-factor model, and the Spanish version yielded a 1-factor solution. On comparing the categorization of items with the analyses of the English and the Dutch version, “Emotional distress,” “Urological and general symptoms,” “Muscle symptoms,” and “Headache/jaw symptoms” were similar factors in both versions, whereas “Sleep disturbance” was a unique factor in the Japanese version. “Emotional distress,” which is the first factor, shared all four items with that of the English version and shared three items with that of the Dutch version. The second factor, “Urological and general symptoms,” encompassed 3 items (11, 21, 25), which loaded on “Urological symptoms” in the English version. In addition, 3 items (9, 22, 25) that loaded on “Physical symptoms” and/or “General disability and physical symptoms” in previous studies were included in this factor. Furthermore, item 23 (Poor memory) loaded sufficiently high on this factor in the present study. The third factor included items 2 and 18, which loaded on “Physical symptoms” in the English version. Because these items referred to muscle problems, this factor was named “Muscle problem.” Item 1 also loaded on this factor. The fourth factor shared all 3 items (4, 10, 19), which loaded on “Headache/jaw symptoms” in the English version; in this version, this factor included unsuitable items (items 7 and 20) which referred to light or smells. The fifth factor encompassed 3 items (1, 8, and 12), which were categorized as physical or emotional symptoms in previous studies; however, due to its reference to sleep, this factor was named “Sleep disturbance”.

A CSI score of 40 out of 100 was the best distinguishing factor between the CSS patient group and a nonpatient comparison sample. Patients with high CSI scores (>40) before knee arthroplasty reported more severe postsurgical pain intensity [57], and patients with CSI scores of >40 before vertebral fusion surgery exhibited higher (i.e. worse) patient-reported disability scores after the surgery [40]. Information regarding patients who score >40 points on the CSI is likely useful for clinicians. The present study found that 10% of the participants were above the moderate severity level (≥40), which was not consistent with previous studies in which 48% of OA patients undergoing total knee arthroplasty and 71% of the patients referred to an interdisciplinary pain clinic showed a CSI score ≥40 [33,53]. The Japanese mean score of the CSI (21.91; SD = 13.31) was lower than the American (52.4; SD = 14.3; 50.7; SD = 13.0) [34,35], Dutch (43.88; SD = 17.67) [36], and Korean (42.4; range 15–80) [57] samples. Previous studies have shown that cultural or ethnic differences influence pain and pain management [58–60]. Cultural differences may continue to contribute to these observed discrepancies. In addition, these discrepancies occurred because characteristics of participants are different, particularly difference of medical condition. In the American and Dutch studies, participants were recruited from a multidisciplinary pain center with complex pain and psychophysiological disorders, including CSSs, or chronic pain disorders (e.g., chronic low back pain, chronic neck pain), and the participants of Korean study were knee OA patients undergoing total knee arthroplasty. In contrast, the present study targeted patients with various musculoskeletal disorders requiring physiotherapy, regardless of duration of pain, region of pain, and type of diagnosis. As many of the participants in this study might experience mild symptoms, more participants showed 40-point or less in CSI, and lesser participants presented previous history of CSSs compared to previous studies. In addition, this fact was supported by a previous study that showed the mean CSI score in patients recruited from the community-based Physiotherapy Program (24.6; SD = 12.0) [38]. Furthermore, our finding that almost 10% of the patients scored high in the CSI-J, supports a previous study that showed 10% of general population complained of symptoms unrelated to organic factors [20].

There were some limitations to the present study. First, we did not measure QST as a direct measure of CS. Further research is needed to examine the validation of the CSI-J by using QST. Second, we did not investigate the sensitivity of the CSI-J to changes in clinical status; hence, we cannot conclude about causation, predictive validity, or response to intervention. Third, the number of CSS was assessed using self-report questionnaire, which potentially involved response bias. Therefore, no patient reported the comorbidity of fibromyalgia because participants might be biased toward responding “no”. Such issues would seem to be appropriate suggestive steps for research on the CSI-J.

## Conclusions

In conclusion, this study reported that the CSI-J was a useful and psychometrically sound tool to assess CSS in Japanese patients with musculoskeletal disorders. The finding of the prevalence rates of CS severity levels in patients with musculoskeletal disorders may help clinicians to decide strategy of treatment.

## Supporting information

S1 Table The Japanese version of CSI. (DOCX)
