# Supplementary material for: Validation of the Japanese version of the Central Sensitization Inventory in patients with musculoskeletal disorders
Source: PLoS One. 2017 Dec 7;12(12):e0188719. doi: 10.1371/journal.pone.0188719 (PMC5720706; doi:10.1371/journal.pone.0188719)
Supplement: S1 Table — (DOCX) [file pone.0188719.s001.docx]

**S1 Table. The Japanese version of the CSI**

**CENTRAL　SENSITIZATION　INVENTORY：PART　A**

名前：　　　　　　　　　　　　　　　　　　　　日付：

**以下の項目について右側の選択肢のうち，最も当てはまるものに○をつけてください．**

| 1.眠りから覚めた時に，疲れていてすっきりしない  感じがする | まったくない | まれにある | ときどき | 頻繁に | いつも |
| --- | --- | --- | --- | --- | --- |
| 2.筋肉に硬さや痛みを感じる | まったくない | まれにある | ときどき | 頻繁に | いつも |
| 3.不安発作がある | まったくない | まれにある | ときどき | 頻繁に | いつも |
| 4.歯を食いしばったり，または歯ぎしりをしたりする | まったくない | まれにある | ときどき | 頻繁に | いつも |
| 5.下痢や便秘の問題を抱えている | まったくない | まれにある | ときどき | 頻繁に | いつも |
| 6.普段の生活での動作を行う上で，助けが必要である | まったくない | まれにある | ときどき | 頻繁に | いつも |
| 7.明るい光に過敏である | まったくない | まれにある | ときどき | 頻繁に | いつも |
| 8.身体を動かすと，すぐに疲れる | まったくない | まれにある | ときどき | 頻繁に | いつも |
| 9.全身のあらゆるところに痛みを感じる | まったくない | まれにある | ときどき | 頻繁に | いつも |
| 10.頭痛がある | まったくない | まれにある | ときどき | 頻繁に | いつも |
| 11.膀胱の不快感と排尿時の灼熱感の両方，または  いずれか一方を感じる | まったくない | まれにある | ときどき | 頻繁に | いつも |
| 12.よく眠れない | まったくない | まれにある | ときどき | 頻繁に | いつも |
| 13.集中することが難しい | まったくない | まれにある | ときどき | 頻繁に | いつも |
| 14.乾燥肌や痒み，発疹などの皮膚の問題がある | まったくない | まれにある | ときどき | 頻繁に | いつも |
| 15.ストレスで身体症状が悪化する | まったくない | まれにある | ときどき | 頻繁に | いつも |
| 16.悲しんだり，または憂鬱な気分になる | まったくない | まれにある | ときどき | 頻繁に | いつも |
| 17.元気が出ない | まったくない | まれにある | ときどき | 頻繁に | いつも |
| 18.首と肩の筋肉が緊張している | まったくない | まれにある | ときどき | 頻繁に | いつも |
| 19.顎に痛みがある | まったくない | まれにある | ときどき | 頻繁に | いつも |
| 20.香水などのある特定の匂いでめまいや吐き気がする | まったくない | まれにある | ときどき | 頻繁に | いつも |
| 21.頻繁に排尿しないといけない | まったくない | まれにある | ときどき | 頻繁に | いつも |
| 22.夜に寝ようとする時，あしに不快感や落ち着かない  感じを感じる | まったくない | まれにある | ときどき | 頻繁に | いつも |
| 23.物事を思い出すことが難しい | まったくない | まれにある | ときどき | 頻繁に | いつも |
| 24.子供の頃に心的外傷(トラウマ)を経験した | まったくない | まれにある | ときどき | 頻繁に | いつも |
| 25.骨盤周辺に痛みがある | まったくない | まれにある | ときどき | 頻繁に | いつも |

**CENTRAL　SENSITIZATION　INVENTORY：PART　B**

名前：　　　　　　　　　　　　　　　　　　　　日付：

**医師から下記の疾患の診断を受けたことがありますか？**

**各診断名の右にある表にチェックをし，診断された年を記入してください**．

|  | いいえ | はい | 診断された年 |
| --- | --- | --- | --- |
| 1. むずむず脚症候群 |  |  |  |
| 1. 慢性疲労症候群 |  |  |  |
| 1. 線維筋痛症 |  |  |  |
| 1. 顎関節症 |  |  |  |
| 1. 片頭痛もしくは緊張性頭痛 |  |  |  |
| 1. 過敏性腸症候群 |  |  |  |
| 1. 化学物質過敏症 |  |  |  |
| 1. 頚部外傷(鞭打ちを含む) |  |  |  |
| 1. 不安発作もしくはパニック発作 |  |  |  |
| 1. うつ病 |  |  |  |
